# Screening for Sleep-Disordered Breathing Risk in Pediatric Dental Care: A Protocol Combining Questionnaire-Based Stratification and Wearable Home Sleep Monitoring

**DOI:** 10.3390/mps9040115

**Published:** 2026-08-01

**Authors:** Parker Norman, Jaclyn Bain, Linda Sangalli, Mitchell Levine, Caroline M. Sawicki

**Affiliations:** 1 Department of Pediatric Dentistry and Dental Public Health, Adams School of Dentistry, University of North Carolina, Chapel Hill, NC 27599, USA; parkern@email.unc.edu (P.N.); jacbain@unc.edu (J.B.); 2 College of Dental Medicine—Illinois, Midwestern University, Downers Grove, IL 60515, USA; lsanga@midwestern.edu; 3 Department of Orthodontics, Adams School of Dentistry, University of North Carolina, Chapel Hill, NC 27599, USA; mlev@unc.edu

**Keywords:** sleep-disordered breathing, pediatric dentistry, obstructive sleep apnea, pediatric sleep questionnaire, home sleep testing, screening, airway assessment, psychosocial functioning

## Abstract

Pediatric sleep-disordered breathing (SDB) is underdiagnosed despite its associations with adverse neurobehavioral, psychosocial, and cardiometabolic outcomes. Pediatric dental providers routinely evaluate craniofacial growth and maintain longitudinal contact with children, yet practical approaches for integrating SDB risk assessment into dental settings remain limited. This protocol describes a prospective, cross-sectional observational study that will enroll 60 school-aged children aged 8–13 years from a university-based pediatric dental clinic and classify them as low-risk or high-risk for SDB using the Pediatric Sleep Questionnaire (PSQ; threshold ≥ 0.33). The primary aim is to examine associations between PSQ-based risk classification and objective physiologic sleep parameters, including the Apnea–Hypopnea Index, Respiratory Disturbance Index, Sleep Apnea Indicator, and Sleep Quality Index, obtained from a U.S. Food and Drug Administration-cleared wearable home sleep monitor—SleepImage Ring (MyCardio LLC, Denver, CO, USA)—worn for a minimum of three consecutive nights. Secondary aims will evaluate associations between SDB risk classification and body mass index, Mallampati score, Brodsky tonsillar grade, and psychosocial functioning (anxiety, depression, perceived stress, and daytime sleepiness). Exploratory craniofacial analyses will be conducted among participants with clinically available lateral cephalometric radiographs. This protocol could position pediatric dental visits as an accessible touchpoint for early identification of children with unrecognized SDB and inform pathways for timely referral. Protocol Version: 1.4, dated 13 May 2026. Trial Registration: ClinicalTrials.gov NCT07581938.

## 1. Introduction

Sleep-disordered breathing (SDB) in children, including obstructive sleep apnea (OSA), produces recurrent intermittent hypoxia and sleep fragmentation that are associated with behavioral dysregulation, neurocognitive deficits, impaired growth, and adverse cardiometabolic outcomes [1,2]. The prevalence of OSA in children is estimated at 1–5%, while habitual snoring affects 10–27% of the pediatric population, making SDB one of the most common sleep disorders in childhood [3,4]. Craniofacial characteristics such as mandibular retrognathia, increased mandibular plane angle, and reduced nasopharyngeal airway dimensions have been associated with upper airway narrowing and increased SDB risk [5,6]. The consequences of delayed identification can be significant, including substantially elevated risk of anxiety (risk ratio [RR] 1.44), depressive disorders (RR 1.71), behavioral disorders (RR 1.31), and learning disorders (RR 1.39) at 1 year among children with delayed versus early surgical intervention [7]. Critically surgery within six months of diagnosis appreciably reduced the risk of these outcomes compared with delayed surgery, suggesting a time-sensitive window for intervention [7]. Despite these well-established associations, pediatric SDB remains under-recognized and under-diagnosed. The American Academy of Pediatrics recommends that all children be screened for snoring at every health maintenance visit [4]. Yet, systematic approaches to early risk identification outside of medical settings remain limited, even though a recent implementation study demonstrated that integrating a structured electronic sleep screener across primary care practices and well-child visits significantly increases sleep disorder diagnoses, polysomnography (PSG) orders, and referrals [8].

Pediatric dental providers routinely evaluate craniofacial growth and development, obtain lateral cephalometric radiographs, and assess clinical features such as high-arched palate, tonsillar hypertrophy, mouth breathing, and dental malocclusion—all of which have been associated with SDB risk and fall outside the typical assessment performed at a well-child visit [6,9,10,11]. Fagundes and Flores-Mir (2022) highlighted that specific craniofacial abnormalities frequently identified by dental professionals have been associated with pediatric OSA, and that dental professionals are well positioned to screen for these features because they assess children more frequently than physicians [11]. Additionally, pediatric dental providers have frequent longitudinal contact with children, providing additional touchpoints for screening that do not exist in the medical system and positioning them well to participate in early screening for SDB risk and to help narrow referrals for full medical assessment.

The Pediatric Sleep Questionnaire (PSQ) is a brief, parent-reported instrument that assesses snoring, breathing-related sleep disturbances, and daytime sleepiness. A cutoff score of ≥0.33 has been validated and widely applied in pediatric populations, including school-aged children, to identify those at increased risk for SDB [12,13,14,15,16]. While the PSQ is not intended as a diagnostic tool, this threshold is commonly used for screening and risk stratification to guide referral for further evaluation [17,18]. A growing body of international evidence supports the feasibility and clinical relevance of SDB screening in dental and orthodontic settings. Across eight studies enrolling 4610 children across 14 countries, PSQ-based screening has consistently identified 7–13% of children at risk for SDB, a rate 1.5 to 2.5 times higher than the approximately 5% prevalence reported in healthy general pediatric populations [19,20,21,22,23,24,25]. The convergence across diverse populations, clinical settings, and geographic regions suggests that dental and orthodontic clinics capture a clinically meaningful proportion of children with unrecognized SDB risk. A systematic review of 12 orthodontic-based studies totaling 3737 participants confirmed that SDB risk is consistently elevated in populations with malocclusions and craniofacial risk markers [26].

While PSG remains the gold standard for diagnosing pediatric OSA, significant barriers limit its use as a corroborative tool in screening-level research. PSG is resource-intensive, costly, and requires specialized equipment and trained personnel. There is a growing body of research documenting that in-laboratory PSG is associated with emotional distress, anxiety, and reduced cooperation in pediatric patients and that these factors can compromise test validity and completion [27,28,29,30,31]. Feasibility studies of home sleep testing in children have demonstrated good technical adequacy, with several reporting greater than 90% of recordings meeting predetermined quality criteria [32]. Advances in wearable sleep technology now complement questionnaire-based screening by enabling objective, home-based assessment of sleep and breathing patterns. FDA-cleared wearable devices can provide indicators of sleep efficiency, cardiopulmonary coupling, and estimated apnea burden across multiple nights, reducing both night-to-night variability and participant burden compared with laboratory-based polysomnography [16,32,33,34]. Wearable home-based sleep monitoring devices can provide physiologic markers of SDB risk without establishing a clinical diagnosis, making them well-suited for screening-level research. A 2026 scoping review of 34 studies on SDB screening in dental settings found that while the PSQ was the most commonly used screening tool, no study examined how screening protocols integrate within routine dental workflows, no study paired questionnaire-based screening with objective psychologic sleep measures, and only one study documented referral outcomes [35].

This prospective, cross-sectional observational study protocol aims to evaluate the feasibility and concordance of integrating questionnaire-based screening, wearable physiologic sleep data, and cephalometric craniofacial measurements in a comprehensive, dental-based approach to identifying children at increased risk for SDB. By combining the structural assessment expertise unique to dental providers with validated screening instruments and objective physiologic measures, this approach addresses gaps in the current screening landscape, complements the symptom = focused screening currently performed in primary care, and may facilitate more targeted referrals for definitive evaluation.

## 2. Materials and Methods

### 2.1. Study Design and Objectives

This is a prospective, cross-sectional observational study conducted at the University of North Carolina (UNC) Adams School of Dentistry (ASOD) Pediatric Dentistry Clinic. Children aged 8–13 years will be classified as low-risk or high-risk for SDB using the PSQ and will undergo anthropometric and airway assessments, validated psychosocial measures, and home sleep monitoring with an FDA-cleared wearable device, manufactured by SleepImage (MyCardio LLC, Denver, CO, USA).

The primary objective is to examine associations between PSQ-based SDB risk classification and objective physiologic sleep parameters obtained through home sleep monitoring. This study is designed as a screening concordance and feasibility investigation, not a diagnostic accuracy study; accordingly, PSG is not included as a reference standard, and findings should be interpreted as characterizing associations between screening-level risk classification and physiologic sleep parameters rather than as establishing diagnostic performance. Secondary objectives include evaluating associations between SDB risk classification and body mass index (BMI), Mallampati score, Brodsky tonsillar grade, and psychosocial functioning, specifically symptoms of anxiety, depression, perceived stress, and daytime sleepiness. An exploratory objective is to characterize craniofacial features associated with SDB risk among participants with clinically available lateral cephalometric radiographs. The inclusion of lateral cephalometric imaging when already clinically available does not exceed minimal risk, as radiation exposure is within the range of standard dental imaging routinely obtained in clinical care.

The planned sample size is 60 participants, with 30 participants classified into each risk group (low-risk vs. high-risk). This sample size is based on the primary aim of comparing physiologic sleep parameters derived from wearable home sleep monitoring between low- and high-risk groups. The minimum sample size per group was determined using the standard formula for comparing two independent group means:

$$n=2{\left(\frac{Z_{1-\alpha /2}+Z_{1-\beta }}{d}\right)}^{2}$$

where $Z_{1-\alpha /2}$ = 1.96 (corresponding to a two-sided significance level of α = 0.05), $Z_{1-\beta }$ = 0.8416 (corresponding to 80% statistical power), and d = 0.80 (Cohen’s d, representing a large standardized effect size). Substituting these values yields

$$n=2{\left(\frac{1.96+0.8416}{0.80}\right)}^{2}=2{\left(\frac{2.8016}{0.80}\right)}^{2}=2{\left(3.502\right)}^{2}=2\left(12.264\right)\approx 25\;\mathrm{per}\;\mathrm{group}$$

This yields a minimum of 25 participants per group. Enrollment was set at 30 per group (*N* = 60 total) to accommodate an anticipated attrition rate of approximately 20%, accounting for incomplete recordings, data quality exclusions, and participant withdrawal while preserving adequate statistical power for the primary analysis.

This study is powered to address the primary aim only. Secondary and exploratory analyses are intended to generate preliminary effect size estimates and variability data to inform future hypothesis-driven studies. Up to 300 individuals may be screened to achieve target enrollment, reflecting the expected distribution of risk classification within the clinic population and anticipated exclusions. A stratified quota sampling approach will be used to ensure balanced representation of each PSQ-defined risk group, a design feature that does not alter the cross-sectional nature of the study but ensures adequate statistical power for between-group comparisons. Participants who discontinue or have incomplete data will not be replaced.

The study was approved by the Institutional Review Board of the University of North Carolina (UNC) at Chapel Hill (IRB#25-3218, 26 January 2026) and registered at ClinicalTrials.gov (NCT07581938, 5 May 2026). Recruitment and follow-up procedures will be conducted between May 2026 and June 2027. This protocol is reported in accordance with the Standard Protocol Items: Recommendations for Interventional Trials (SPIRIT) 2025 statement [36] and adapted accordingly for this prospective, cross-sectional observational study. A completed SPIRIT 2025 checklist is provided as Supplementary Table S1.

### 2.2. Study Setting and Recruitment Workflow

Participants will be recruited from the Pediatric Dentistry Clinic at the UNC ASOD, a university-based practice providing comprehensive oral healthcare for children and adolescents. Recruitment will occur among patients presenting for new patient examinations, periodic recall visits, and other routine dental appointments.

Research personnel will review upcoming clinic schedules and electronic health records under an Institutional Review Board (IRB)-approved limited Health Insurance Portability and Accountability Act (HIPAA) waiver to identify children within the eligible age range and assess preliminary eligibility. Parents or legal guardians of potentially eligible participants may be contacted by telephone prior to the scheduled dental appointment to introduce the study and assess interest. Families who cannot be reached by telephone or who prefer to discuss participation in person may be approached at the time of their child’s scheduled visit. Following confirmation of eligibility and informed consent and assent completion by trained study personnel, participants will complete baseline study assessments either immediately before or after their dental appointment to minimize additional visits.

### 2.3. Screening and Eligibility

Children meeting all inclusion criteria and none of the exclusion criteria will be eligible for enrollment. The age range of 8–13 years was selected to capture a developmental period when SDB may influence craniofacial growth and psychosocial functioning while also allowing reliable completion of self-report measures.

Eligible participants will be children aged 8–13 years receiving care at the UNC ASOD Pediatric Dentistry Clinic with an American Society of Anesthesiologists (ASA) Physical Status Classification of I or II. Participants must be able to read and understand English or Spanish, provide assent with parental or guardian consent, and have access to a smartphone compatible with the home sleep monitoring application.

Participants will be excluded if they have a prior diagnosis of SDB or OSA, history of adenotonsillectomy, craniofacial syndromes or conditions known to substantially alter craniofacial morphology, prior or current orthodontic treatment, or prior orthognathic surgery. These exclusion criteria were selected to reduce heterogeneity in upper airway anatomy and SDB risk factors that could confound interpretation of physiologic sleep and craniofacial outcomes.

### 2.4. PSQ-Based Risk Classification

SDB risk will be assessed using the PSQ, a validated caregiver-completed screening instrument widely used in pediatric research and clinical settings [12]. PSQ scores will be calculated by dividing the number of affirmative responses by the total number of completed items. Participants will be classified as low-risk (PSQ score < 0.33) or high-risk (PSQ score ≥ 0.33) based on the established threshold for pediatric SDB risk stratification. Detailed administration procedures, scoring algorithms, handling of missing data, interpretation thresholds, and psychometric properties for the PSQ are provided as Supplementary Materials [12,37,38,39,40,41,42,43].

The PSQ is used in this study to stratify participants by SDB risk rather than to establish a clinical diagnosis. No diagnostic determinations will be made based on questionnaire responses alone.

### 2.5. Home Sleep Monitoring Methods

Objective physiologic sleep data will be collected using the SleepImage Ring, an FDA-cleared wearable home sleep monitoring device, and analyzed using the FDA-cleared SleepImage System (Version 2.28, MyCardio LLC, Denver, CO, USA). Following completion of baseline assessments, participants will receive the device along with standardized instructions for use, charging, and smartphone application synchronization. Participants and their parents or guardians will be instructed to maintain usual sleep routines throughout the monitoring period.

Participants will wear the device during sleep for a minimum of three consecutive nights. To accommodate scheduling challenges, missed nights, and device return logistics, participants will be permitted up to three weeks to complete the required monitoring period. Physiologic sleep data will be collected passively during sleep and transmitted through a secure, HIPAA-compliant platform.

Primary physiologic sleep outcomes will include the cardiopulmonary coupling (CPC) software-derived Apnea-Hypopnea Index (sAHI), software-derived Respiratory Disturbance Index (sRDI), Sleep Apnea Indicator (SAI), and Sleep Quality Index (SQI), provided as continuous data. To ensure adequate data quality, only recordings with an average signal quality of at least 50% and a minimum sleep duration of four hours per night will be included in analyses. A minimum of two qualifying recording nights will be required for a participant’s data to be included in the primary analysis. Participants with only one qualifying night will be excluded from the primary analysis but will be retained for sensitivity analyses.

Adherence will be supported through brief telephone check-ins approximately one and two weeks following device distribution to address technical questions and encourage compliance. Home-based monitoring was selected over laboratory polysomnography to enable assessment within the participant’s natural sleep environment while minimizing burden and supporting feasibility within a pediatric dental setting.

### 2.6. Clinical and Psychosocial Assessments

Clinical, anthropometric, and psychosocial assessments will be completed during the baseline study visit, prior to initiation of home sleep monitoring. Height and weight will be obtained using standard clinical procedures and used to calculate body mass index (BMI, kg/m^2^). Airway assessments will include the Mallampati classification and Brodsky tonsillar grading scale, both of which have been associated with upper airway obstruction and SDB risk in pediatric populations. Two calibrated examiners will independently perform Mallampati and Brodsky assessments blinded to each other’s scores and to the PSQ results. Discrepancies will be resolved by consensus review. All clinical assessments will be performed by trained study personnel using standardized procedures.

Psychosocial functioning will be assessed using validated self-report instruments administered electronically. Symptoms of anxiety and depression will be measured using the Revised Children’s Anxiety and Depression Scale-Short Version (RCADS-25), a 25-item self-report measure scored on a 4-point Likert scale (0 = never to 3 = always), yielding separate anxiety and depression subscale scores with higher values indicating greater symptom severity [37,38]. Perceived stress will be assessed using the Perceived Stress Scale for Children (PSS-C), a 13-item self-report measure scored on a 4-point Likert scale, with higher total scores reflecting greater perceived stress [39]. Daytime sleepiness will be evaluated using the Sleepiness Scale of the Children’s Report of Sleep Patterns (CRSP-S), a validated self-report measure of daytime sleepiness for school-aged children with demonstrated reliability and construct validity [40].

Detailed administration procedures, scoring algorithms, handling of missing data, interpretation thresholds, and psychometric properties for each psychosocial instrument (RCADS-25, PSS-C, CRSP-S) are provided as Supplementary Materials.

### 2.7. Exploratory Craniofacial Assessment

Exploratory craniofacial analyses will be conducted among participants with clinically available lateral cephalometric radiographs obtained as part of routine dental or orthodontic care. No radiographs will be obtained specifically for research purposes, and study participation will not require the availability of a cephalometric radiograph. Clinically obtained lateral cephalometric radiograph within six months prior to or within six months after the baseline study visit or enrollment will be used when available in the electronic medical record. For participants with multiple available radiographs, the image obtained closest to the baseline study visit will be selected for analysis.

Cephalometric measurements will be obtained using standardized landmark identification and tracing procedures by calibrated study personnel. Variables of interest will include dental and skeletal planar values and skeletal vertical and anterior–posterior values relevant to upper airway patency. Because cephalometric imaging will only be available for a subset of participants and was not obtained under a standardized research imaging protocol, these analyses are considered exploratory and hypothesis-generating.

### 2.8. Outcome Measures

#### 2.8.1. Primary Outcome

The primary outcome will be physiologic sleep parameters obtained from the wearable home sleep monitoring device, including the sAHI, sRDI, SAI, and SQI. The sAHI is an automated, FDA-cleared measure of apnea and hypopnea events per hour that quantifies respiratory disturbance burden during sleep, which will be reported at both ≥3% and ≥4% oxygen desaturation thresholds with additional obstructive and central subtype differentiation. The total AHI at 3% desaturation (AHI3%) is consistent with the American Academy of Sleep Medicine (AASM) recommended scoring rule [44] and the total AHI at 4% desaturation (AHI4%) corresponds to the Centers for Medicare & Medicaid Services (CMS) reimbursement criterion [45]. The sRDI captures apnea and hypopnea events as well as arousal-related respiratory disturbances that may not be associated with oxygen desaturation but nonetheless contribute to sleep fragmentation. This broader scope may provide a more comprehensive assessment of respiratory disturbance burden than indices based solely on desaturation-linked events [46,47,48]. Because the wearable device estimates respiratory event indices using CPC analysis rather than direct airflow measurement, device-derived values are not equivalent to PSG-scored AHI; this distinction will be acknowledged in the interpretation of the results. This study is designed as a screening concordance and feasibility investigation, not a diagnostic accuracy study; accordingly, PSG is not included as a reference standard, and findings should be interpreted as characterizing associations between screening-level risk classification and physiologic sleep parameters rather than as establishing diagnostic performance.

In addition to respiratory event indices, two composite cardiopulmonary coupling-derived metrics will be examined. The SAI is a CPC-derived metric that detects cyclic heart rate variations (bradycardia during apneic events followed by relative tachycardia upon breathing resumption) reflecting the autonomic cardiac response to intermittent hypoxemia during unstable breathing. This cardiac-based approach may be particularly relevant in pediatric populations, where apneic events are often shorter and may not meet conventional oximetry desaturation thresholds [49,50]. The SQI is a composite metric derived from CPC analysis that integrates biomarkers of sleep stability, duration, and fragmentation to characterize overall sleep health. Age- and sex-specific normative values have been established for the pediatric age range relevant to this study [34,51].

For participants with two or three qualifying nights, physiological sleep outcomes (CPC, sAHI, sRDI, SAI, and SQI) will be averaged across qualifying nights to generate participant-level outcome measures and compared between PSQ-based SDB risk groups.

#### 2.8.2. Secondary Outcomes

Secondary outcomes include anthropometric, airway, and psychosocial measures. BMI will be evaluated as a continuous variable and categorized using age- and sex-specific CDC percentiles (normal weight, overweight, obese). Mallampati classification (Class I-IV) and Brodsky tonsillar grade (0–4) will be analyzed as ordinal variables.

Psychosocial outcomes include anxiety and depression symptom scores from the RCADS-25, perceived stress scores from the PSS-C, and daytime sleepiness scores from the CRSP-S. For the RCADS-25, both total scores and subscale scores (anxiety, depression) will be examined, with T-scores ≥ 65 indicating borderline clinical significance and ≥70 indicating clinical significance. PSS-C total scores will be analyzed as a continuous variable, with higher scores indicating greater perceived stress. CRSP-S scores will be analyzed as a continuous variable, with higher scores indicating greater daytime sleepiness.

#### 2.8.3. Exploratory Outcomes

Exploratory outcomes include craniofacial and airway-related measurements derived from clinically available lateral cephalometric radiographs. Craniofacial skeletal anterior–posterior, dental anterior–posterior, craniofacial skeletal vertical, dental vertical, and soft tissue profile values are of particular interest due to their relevancy to upper airway patency. These measurements will be summarized descriptively and compared between risk groups. Because imaging is available only for a subset of participants, these analyses are intended to generate preliminary effect size estimates and inform the design of future hypothesis-driven investigations.

### 2.9. Statistical Analysis

Descriptive statistics will be used to summarize participant characteristics, clinical measures, psychosocial outcomes, and physiologic sleep parameters. Continuous variables will be reported as means and standard deviations or medians and interquartile ranges depending on distributional characteristics assessed by Shapiro–Wilk tests, while categorical variables will be summarized using frequencies and percentages.

For the primary analysis, physiologic sleep parameters (sAHI, sRDI, SAI, SQI) will be averaged across qualifying recording nights for each participant and compared between PSQ-based risk groups using independent-samples *t*-tests or Mann–Whitney U tests, as appropriate. Night-to-night reliability of physiologic sleep parameters will be assessed using intraclass correlation coefficients (ICCs). Effect sizes will be reported using Cohen’s d and interpreted according to conventional thresholds, with values of approximately 0.20, 0.50, and 0.80 representing small, medium, and large effects, respectively. Sensitivity analyses will be conducted by (1) restricting analyses to participants with three qualifying recording nights; (2) repeating analyses using only the first qualifying night for each participant; and (3) exploring the inclusion of participants with only one qualifying recording night using the single available recording. Multivariable linear regression models will be used to examine associations between SDB risk classification and physiologic sleep measures while adjusting for prespecified covariates including age, sex, and BMI percentile. Additional covariates (asthma, ADHD) will be included if baseline group differences are identified or if they demonstrate meaningful associations with primary outcomes.

Secondary analyses will evaluate associations between SDB risk classification and psychosocial measures (RCADS-25 scores, PSS-C scores, CRSP-S scores) and BMI using linear regression models. Ordinal outcomes (Mallampati classification, Brodsky tonsillar grade) will be analyzed using Mann–Whitney U tests or ordinal logistic regression, as appropriate.

Exploratory craniofacial analyses will be conducted among the subset of participants with available lateral cephalometric radiographs. Cephalometric measurements will be summarized descriptively and compared between risk groups using independent-samples *t*-tests or Mann–Whitney U tests. Given the anticipated smaller sample size and exploratory nature of these analyses, findings will be reported as effect size estimates with 95% confidence intervals to inform future study design.

No formal adjustment for multiple comparisons will be applied to secondary and exploratory analyses, as these are intended to generate preliminary estimates rather than confirmatory conclusions. All statistical analyses will be performed using SPSS (v. 31, IBM SPSS Statistics, IBM Corp., Armonk, NY, USA). Statistical significance will be evaluated using two-sided tests with an α level of 0.05.

### 2.10. Data Management and Confidentiality

Study data will be collected and managed using the Research Electronic Data Capture (REDCap), a secure, web-based data management platform hosted by UNC. REDCap provides audit trails for data entry and modification, role-based access controls, and encrypted data storage. Built-in validation features, including range checks and required field prompts, will be used to minimize data entry errors. Periodic data quality reviews will be conducted by study personnel to identify missing or inconsistent entries.

Participants will be assigned unique study identification numbers at enrollment. A master linking file connecting study identification numbers to participant identifiers will be stored separately from study data on a password-protected, encrypted institutional server accessible only to authorized study personnel approved by the IRB. All study data, including questionnaire responses, clinical assessments, and physiologic sleep data, will be linked using study identification numbers rather than personal identifiers.

Physiologic sleep data collected through the wearable home sleep monitoring device will be transmitted through a secure, HIPAA-compliant platform and incorporated into the REDCap database following completion of the monitoring period and quality review. All electronic data will be stored on secure institutional servers in accordance with UNC policies governing research involving human participants.

No formal interim analyses are planned. As this is a non-interventional, observational feasibility study with no therapeutic interventions, formal stopping rules for efficacy or futility are not applicable. The principal investigator will have access to all accumulating data and will make the final decision regarding study continuation or termination, in consultation with the investigative team and the IRB as appropriate.

Research records will be retained for a minimum of 3 years following study completion, in accordance with UNC Office of Human Research Ethics policy and Title 45, Part 46 of the Code of Federal Regulations (45 CFR 46.115). Signed HIPAA authorizations will be retained for a minimum of 6 years. De-identified data may be made available to qualified investigators upon reasonable request and in accordance with applicable institutional and regulatory requirements. All study procedures will be conducted in accordance with applicable federal regulations, institutional policies, and the approved Institutional Review Board protocol.

## 3. Procedure

### 3.1. Participant Workflow and Study Timeline

**Figure 1:**
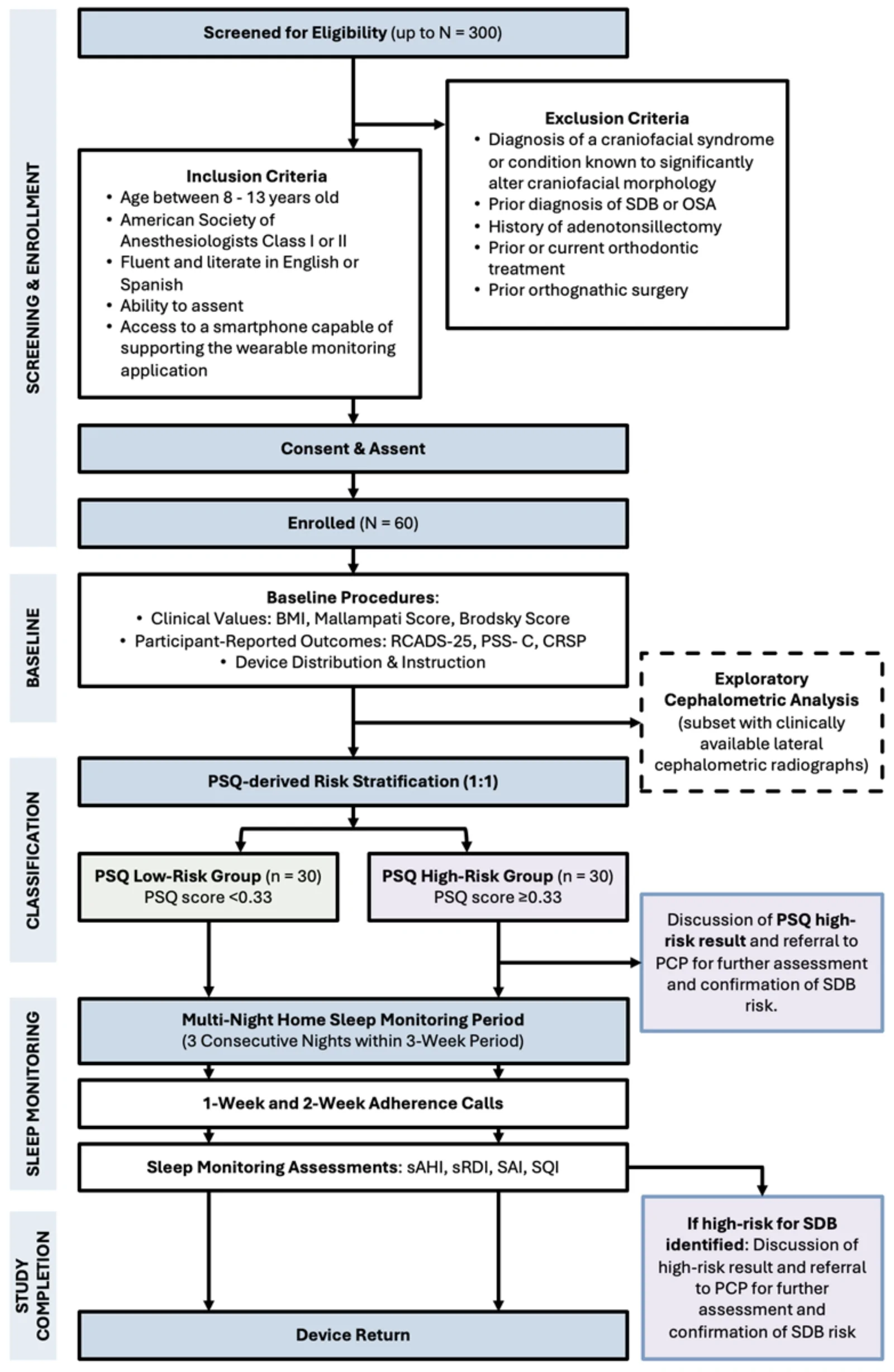
Participant workflow for multimodal sleep-disordered breathing risk assessment. Following screening and enrollment, participants complete baseline clinical, airway, and psychosocial assessments and are classified as low-risk or high-risk for SDB based on Pediatric Sleep Questionnaire (PSQ) scores. Participants then complete a minimum of three consecutive nights of home sleep monitoring using the SleepImage Ring, with adherence check-ins at approximately one and two weeks. Study participation concludes after device return and verification of data completeness. Exploratory cephalometric analyses are performed for the subset of participants with clinically available lateral cephalometric radiographs. Abbreviations: PSQ = Pediatric Sleep Questionnaire; RCADS-25 = Revised Children’s Anxiety and Depression Scale–Short Version; PSS-C = Perceived Stress Scale for Children; CRSP-S = Children’s Report of Sleep Patterns Sleepiness Scale; BMI = body mass index; PCP = Primary Care Provider; sAHI = software-derived Apnea-Hypopnea Index; sRDI = software-derived Respiratory Disturbance Index; SAI = Sleep Apnea Indicator; SQI = Sleep Quality Index; SDB = sleep-disordered breathing.

This study consists of four phases: screening and enrollment, baseline assessment, home sleep monitoring, and study completion. Potentially eligible participants are identified through a review of pediatric dentistry clinic schedules and electronic health records. Following confirmation of eligibility and completion of informed consent and assent procedures, participants complete baseline clinical, airway, and psychosocial assessments as described in Section 2.4 and Section 2.6. Parents or guardians complete the PSQ, and participants are classified as low- or high-risk for sleep-disordered breathing based on established scoring thresholds (Section 2.4). Following completion of baseline assessments, participants receive a wearable home sleep monitoring device and standardized instructions for use (Section 2.5). Participants are instructed to wear the device during sleep for a minimum of three consecutive nights within the following three weeks. Brief telephone contacts are conducted approximately one and two weeks following device distribution to support adherence and address technical questions. Study participation concludes after completion of home sleep monitoring, device return, and final review of study procedures. Figure 1 illustrates the participant workflow, and Table 1 summarizes the schedule of study assessments.

### 3.2. Baseline Assessment Procedures

All baseline assessments are completed during a single study visit coinciding with the participant’s scheduled dental appointment. The visit proceeds in the following sequence: (1) confirmation of parental consent, HIPAA authorization, child assent, and eligibility; (2) collection of demographic and medical history information through medical record review and participant report; (3) parent or guardian completion of the PSQ, which is used to classify participants into low- and high-risk SDB groups; (4) anthropometric measurements (height, weight, BMI calculation); (5) intraoral airway assessments (Mallampati classification and Brodsky tonsillar grading) performed by trained and blinded study personnel; and (6) electronic administration of psychosocial questionnaires (RCADS-25, PSS-C, CRSP Sleepiness Scale) completed by the participant. Detailed descriptions of each assessment instrument, scoring procedures, and psychometric properties are provided in Section 2.4 and Section 2.6. Following completion of all baseline assessments, participants receive the home sleep monitoring device and standardized instructions for use.

### 3.3. Home Sleep Monitoring Procedures

Following device distribution, participants and their parents or guardians are oriented to the SleepImage Ring, including device placement, nightly charging, and synchronization with the accompanying smartphone application. Participants are instructed to maintain their usual sleep routines throughout the monitoring period. To accommodate scheduling conflicts, missed nights, or device return delays, participants are permitted up to three weeks to complete the required minimum of three consecutive nights of monitoring. Research personnel conduct brief adherence check-in telephone calls approximately one and two weeks after device distribution to address technical concerns, encourage compliance, and answer participant questions. No additional study assessments are administered during these calls. Following completion of home sleep monitoring by the first 10 participants, a planned data quality review will be conducted to evaluate potential technical or operational issues, such as device functionality, data completeness, signal quality, successful data transmission, and adherence to the monitoring protocol. Any necessary procedural modifications will be documented prior to continuation of enrollment. Following completion of monitoring, physiologic sleep data will undergo quality assessment for signal quality and completeness prior to extraction of study outcomes; only recordings meeting prespecified quality criteria on at least two nights (average signal quality ≥ 50%, minimum sleep duration ≥ 4 h per night) are included in analyses. Device specifications and physiologic sleep outcome variables are described in Section 2.5.

### 3.4. Adverse Event Monitoring and Study Compliance

Research personnel will document any adverse events or participant concerns reported during the study period. Anticipated risks associated with study participation are minimal. Potential risks include mild skin irritation or discomfort from wearing the SleepImage Ring during sleep, brief discomfort during intraoral airway assessment, and the possibility of emotional discomfort when completing psychosocial questionnaires. Participants and their parents or guardians will be informed of these potential risks during the consent and assent process and may withdraw from the study at any time without affecting their clinical care. Any unanticipated adverse events will be reported to the IRB in accordance with institutional policies and applicable regulatory requirements.

Study participation concludes after completion of the home sleep monitoring period, return of the wearable device, and verification of data completeness. No diagnostic determinations or therapeutic interventions will be delivered as part of the study protocol, and study participation will not alter the participants’ routine dental care. Consistent with established ethical principles for the return of clinically significant research findings, parents/guardians of children identified as high-risk on the PSQ will be informed of the screening result and provided with educational materials about pediatric SDB. Notification will follow a standardized script that clearly distinguishes screening-level risk identification from clinical diagnosis, emphasizes that study findings do not constitute a medical evaluation, and recommends follow-up with the child’s primary care provider or a sleep medicine specialist for further assessment. The notification script and accompanying parent-facing educational materials are maintained as part of the study’s IRB-approved procedures. This approach is supported by evidence that parental awareness of SDB risk facilitates timely referral and evaluation, and that structured screening with parental notification significantly increases rates of sleep disorder identification and appropriate clinical follow-up.

Protocol oversight is maintained through regular communication among the investigative team, including a review of enrollment progress, retention, participant-reported concerns, and protocol deviations. Given the observational, non-interventional design and anticipated small sample size, a formal Data Safety Monitoring Board was not convened. Any protocol modifications will be submitted to the IRB for approval prior to implementation.

## 4. Expected Results

This study is designed to characterize associations between questionnaire-based SDB risk classification and physiologic, clinical, and psychosocial measures in a pediatric dental population. Expected outcomes are grounded in the existing literature linking pediatric SDB with adverse sleep, airway, anthropometric, and psychosocial characteristics.

Physiologic Sleep Outcomes. Children classified as high-risk for SDB based on PSQ scores are expected to demonstrate higher AHI, RDI, and SAI values, as well as lower SQI scores, compared with children classified as low-risk. In the original PSQ validation study, the 0.33 threshold on the sleep-related breathing disorder subscale produced a sensitivity of 0.81–0.85 and a specificity of 0.87 for SDB confirmed by PSQ [12]. These findings support the expectation that PSQ-based risk stratification will correspond with objective physiologic differences detected by home sleep monitoring. If these associations are not observed, this may reflect differences in the sensitivity of wearable home monitoring compared with laboratory-based PSG or the influence of night-to-night variability in pediatric sleep parameters.

Airway and Anthropometric Outcomes. Higher SDB risk is expected to be associated with greater BMI, larger tonsillar size as measured by the Brodsky scale, and higher Mallampati classifications. Adenotonsillar hypertrophy is the most commonly recognized risk factor for pediatric SDB, and the prevalence of OSA among children with obesity is estimated at 45% compared with 9% among children with healthy weight, with a 1-unit increase in BMI standard deviation score increasing the odds of OSA by a factor of 1.9 independent of age, sex, and tonsillar hypertrophy [52,53,54,55]. These associations have been demonstrated across diverse pediatric populations and are expected to be replicated within this dental clinic sample.

Psychosocial Outcomes. Children classified as high-risk for SDB are expected to demonstrate greater symptoms of anxiety, depression, perceived stress, and daytime sleepiness compared with children classified as low-risk. SDB symptoms have been positively associated with depression, anxiety, attention problems, and reduced adaptability in school-aged children, and a recent meta-analysis estimated an overall 28% prevalence of depression among children with or at high risk for OSA, approximately two-fold higher than among children without OSA [56,57,58]. School-aged children with OSA have also demonstrated significantly higher scores on the Spence Children’s Anxiety Scale and Children’s Depression Inventory compared with controls, with symptom severity correlating with disease duration and hypoxia burden [59]. The inclusion of validated psychosocial instruments in this protocol allows for characterization of these associations within a dental setting where such measures are not routinely collected.

Exploratory Craniofacial Outcomes. Among participants with clinically available lateral cephalometric radiographs, exploratory analyses may identify craniofacial characteristics associated with increased SDB risk. A systematic review and meta-analysis found that children with OSA tend to present with mandibular retrognathia (reduced SNB), increased sagittal skeletal jaw discrepancy (increased ANB), and a steeper mandibular plane angle (increased FH-MP) compared with controls [5]. Additional features described in the literature include increased lower anterior facial height, narrower maxillary arch width, and reduced anteroposterior dimensions of the bony nasopharynx [11,60]. Because radiographs are available only for a subset of participants and are obtained through routine clinical care rather than a standardized research imaging protocol, this subset likely represents an orthodontically indicated subpopulation enriched for skeletal and dental anomalies rather than a random sample of the broader pediatric dental clinic population. Accordingly, cephalometric findings may not be generalizable to all children seen in pediatric dental settings, and between-group differences should be interpreted with this selection bias in mind. These analyses are intended to generate preliminary data and inform future hypothesis-driven investigations.

Feasibility and Methodological Implications. Beyond clinical outcomes, this protocol is expected to demonstrate the feasibility of incorporating questionnaire-based risk stratification, wearable home sleep monitoring, airway assessment, and psychosocial evaluation into a pediatric dental research setting. Practical outcomes of interest include participant recruitment yield, device adherence rates, data completeness, and acceptability of study procedures within the clinic workflow. These feasibility data will inform the design and implementation of future larger-scale investigations examining the role of dental providers in early identification of children at increased risk for SDB. Several methodological limitations should be acknowledged. This study is designed as a screening concordance and feasibility investigation, not a diagnostic accuracy study; accordingly, polysomnography is not included as a reference standard, and findings should be interpreted as characterizing associations between screening-level risk classification and physiologic sleep parameters rather than as establishing diagnostic performance. Additionally, participants contributing only two qualifying recording nights may have greater variability in physiologic sleep estimates than those with three nights, given documented night-to-night variability in pediatric respiratory parameters [61,62]. The reliability of physiologic sleep measures across qualifying recording nights will be evaluated using ICCs. Planned sensitivity analyses restricting analyses to participants with three qualifying nights and comparing results using a single-night recording approach will evaluate the influence of recording duration on study findings. Exploratory analyses including participants with only one qualifying recording night will further assess whether exclusion of incomplete recordings influences study conclusions. The minimum number of recording nights needed for stable cardiopulmonary coupling-derived estimates in pediatric populations has not been established and warrants investigation in future studies.

## Figures and Tables

**Table 1:** Schedule of study assessments by study phase.

Study Timepoint	Study Activity	Screening Enrollment	Visit #1 (Baseline)	Home Sleep Monitoring	Visit #2
Enrollment	Eligibility screening	X			
	EHR review	X			
	Informed consent	X			
	Child assent	X			
	HIPAA authorization	X			
	Demographics & Medical history		X		
Clinical Values	BMI		X		
	Mallampati score		X		
	Brodsky score		X		
Classification	PSQ (Parent-reported)		X		
	PSQ scoring risk classification		X		
	Parental notification of PSQ risk status		X		
Participant-reported Outcomes	RCADS-25		X		
	PSS-C		X		
	CRSP-S		X		
Device Distribution	Wearable device fitting & instructions		X		
Follow-Up	Adherence Check-In Calls (Weeks 1 and 2)			X	
Sleep Parameters	sAHI			X	
	sRDI			X	
	SAI			X	
	SQI			X	
	Parental notification of device-derived risk status			X	
Exploratory Outcomes	Cephalometric Analysis		X *		
Study Completion	Device return				X

X indicates at what study phase each assessment is planned to be completed. * Analyzed for subset of participants with clinically available lateral cephalometric radiographs (within six months pre- or post-baseline visit/enrollment); no research radiographs obtained. Abbreviations: BMI = body mass index; PSQ = Pediatric Sleep Questionnaire; RCADS-25 = Revised Children’s Anxiety and Depression Scale–Short Version; PSS-C = Perceived Stress Scale for Children; CRSP-S = Children’s Report of Sleep Patterns Sleepiness Scale; sAHI = software-derived Apnea-Hypopnea Index; sRDI = software-derived Respiratory Disturbance Index; SAI = Sleep Apnea Indicator; SQI = Sleep Quality Index.

## Data Availability

The original contributions presented in this study are included in the article/Supplementary Material. Further inquiries can be directed to the corresponding author.

## Supplementary Materials

The following supporting information can be downloaded at: https://www.mdpi.com/article/10.3390/mps9040115/s1, Table S1: SPIRIT 2025 checklist. Supplementary Material: Questionnaire administration, scoring, and interpretation guide (PSQ, RCADS-25, PSS-C, CRSP-S) [12,37,38,39,40,41,42,43].

## Abbreviations

The following abbreviations are used in this manuscript:

AASM	American Academy of Sleep Medicine
ADHD	Attention-Deficit/Hyperactivity Disorder
AHI	Apnea-Hypopnea Index
ASA	American Society of Anesthesiologists
ASOD	Adams School of Dentistry
BMI	body mass index
CFR	Code of Federal Regulations
CHAT	Childhood Adenotonsillectomy Trial
CMS	Centers for Medicare & Medicaid Services
CPC	cardiopulmonary coupling
CVHR	cyclic variation in heart rate
FDA	U.S. Food and Drug Administration
HIPAA	Health Insurance Portability and Accountability Act
IRB	Institutional Review Board
ODD	Oppositional Defiant Disorder
OSA	obstructive sleep apnea
PSG	polysomnography
PSQ	Pediatric Sleep Questionnaire
RDI	Respiratory Disturbance Index
RR	risk ratio
sAHI	software-derived Apnea-Hypopnea Index
SAI	Sleep Apnea Indicator
SD	standard deviation
SDB	sleep-disordered breathing
SQI	Sleep Quality Index
sRDI	software-derived Respiratory Disturbance Index
UNC	University of North Carolina
