# Supplementary material for: Screening for Sleep-Disordered Breathing Risk in Pediatric Dental Care: A Protocol Combining Questionnaire-Based Stratification and Wearable Home Sleep Monitoring
Source: Methods Protoc. 2026 Aug 1;9(4):115. doi: 10.3390/mps9040115 (PMC13516765; doi:10.3390/mps9040115)
Supplement: Supplementary file 1 [file mps-09-00115-s001.zip › SDB Protocol Supplementary Materials v2.pdf]

Screening for Sleep-Disordered Breathing Risk in Pediatric Dental Care: A Protocol Combining Questionnaire-Based Stratification and Wearable Home Sleep Monitoring – *Supplementary Material*

This supplementary material provides detailed information on the administration, scoring, and interpretation of the four validated questionnaire instruments used in this study protocol. These instruments are described in Sections 2.4 and 2.6 of the main manuscript and include the Pediatric Sleep Questionnaire (PSQ), the Revised Children's Anxiety and Depression Scale 25-Item Version (RCADS-25), the Perceived Stress Scale for Children (PSS-C), and the Children's Report of Sleep Patterns – Sleepiness Scale (CRSP-S). For each instrument, standardized procedures for administration, data processing, scoring algorithms, clinical interpretation thresholds, psychometric properties, and licensing requirements are provided to support methodological transparency and facilitate replication of this protocol in other pediatric dental or clinical research settings. Investigators seeking to implement this screening protocol should consult the original validation references cited herein and obtain appropriate permissions prior to use.

Questionnaire Administration

A. Pediatric Sleep Questionnaire (PSQ)

1. *General Description*
    a. Full name: Pediatric Sleep Questionnaire (PSQ) – Sleep-Related Breathing Disorder Scale (PSQ-SRBD)
    b. Original author(s): Chervin RD et al. (2000) [12]
    c. Construct measured: Symptoms of sleep-related breathing disorders, including snoring, daytime sleepiness, and inattention/hyperactivity behaviors
    d. Target population: Children (typically ages 2–18 years)
    e. Respondent: Parent/caregiver (proxy report)
    f. Number of items: 22 items
    g. Domains/subscales:
        i. Snoring
        ii. Sleepiness
        iii. Inattention/Hyperactivity
        iv. Other sleep-related breathing items
    h. Recall period: Typical/habitual sleep behaviors (no fixed timeframe specified)
2. *Administration*

   a. Format: Paper-and-pencil or electronic questionnaire completed by parent/caregiver
   b. Estimated completion time: ~5 minutes
   c. Response options: "Yes" (scored as 1), "No" (scored as 0), "Don't know" (treated as missing)
   d. Special instructions: Questionnaire is answered independently by the caregiver with research staff available for questions.
   e. Training requirements: None; can be administered by research staff with minimal instruction
3. *Data Processing / Scoring*
   a. Scoring algorithm: The SRBD score is calculated as the proportion (mean) of positive ("Yes") responses among non-missing items:
   b. SRBD Score = (Number of "Yes" responses) / (Number of items answered "Yes" or "No")
   c. Score range: 0.00 to 1.00
   d. Handling of missing data: Items answered as "Don't know" are excluded from both the numerator and denominator. Maximum allowable proportion of missing items for a valid score not strictly established. A minimum completion threshold of ≥ 18 of 22 items (≥ 80%) was applied; questionnaires with > 4 missing items were treated as invalid to maximize data retention while ensuring score validity [41].
   e. Subscale scoring: Each subscale (Snoring, Sleepiness, Inattention/Hyperactivity) is scored as the mean of its constituent items using the same proportional method.
   f. Reverse scoring: None required
   g. Scoring time: ~1 minute (manual); instantaneous if automated
4. *Interpretation*
   a. Clinical cutoff: A score ≥ 0.33 on the 22-item SRBD scale is considered positive/suggestive of sleep-disordered breathing
   b. Diagnostic performance (against polysomnography-defined OSA):
      i. Sensitivity: ~81%
      ii. Specificity: ~87%
      iii. AUC: 0.85
   c. Psychometric properties:
      i. Internal consistency: Cronbach's α = 0.88 (for the 22-item SRBD scale)
      ii. Test-retest reliability: $r = 0.75$
      iii. Construct validity: Significant correlations with polysomnographic indices (AHI, oxygen desaturation)
   d. Direction of scoring: Higher scores indicate greater symptom burden / higher likelihood of sleep-disordered breathing

5. *Licensing/permissions*: The Pediatric Sleep Questionnaire (PSQ) is copyrighted by the University of Michigan and managed via MAPI Research Trust ePROVIDE (Lyon, France), requiring formal requests for distribution, academic research use at no charge, and paid licenses or user fees for commercial or funded applications.

B. Revised Children's Anxiety and Depression Scale 25-Item Version (RCADS-25)

1. *General Description*
   a. Full name: Revised Children's Anxiety and Depression Scale 25-Item Version (RCADS-25)
   b. Original author(s): Ebesutani C. et al. (2012) [37]
   c. Construct measured: Anxiety and depressive symptoms in children and adolescents
   d. Target population: Children and adolescents (ages ~8-18 years; Grades 3-12)
   e. Respondent: Child/adolescent self-report (a parent version, RCADS-25-P, is also available)
   f. Number of items: 25
   g. Subscales:
      i. Anxiety Total (15 items)
      ii. Depression (Major Depressive Disorder) Total (10 items)
      iii. Total Anxiety and Depression Score (all 25 items)
   h. Recall period: No fixed timeframe specified
2. *Administration*
   a. Format: Paper-and-pencil or electronic self-report
   b. Estimated completion time: ~5-10 minutes
   c. Response options: 4-point Likert scale: 0 = "Never," 1 = "Sometimes," 2 = "Often," 3 = "Always"
   d. Special instructions: Questionnaire is answered independently by the participant with research staff available for questions.
   e. Training requirements: Minimal; can be administered by trained research assistants
3. *Data Processing / Scoring*
   a. Scoring algorithm:
      i. Sum raw scores for each subscale (Anxiety: 15 items, range 0-45; Depression: 10 items, range 0-30; Total: 25 items, range 0-75)
      ii. Convert raw scores to T-scores using age- and gender-based normative tables (available at https://rcads.ucla.edu/scoringoverview)
   b. Handling of missing data: To address missing raw data, scores can be prorated for the existing items within their respective scales, provided they meet specific completeness thresholds. It is recommended that the Total

Depression subscale be left unscored if it lacks more than two items, and similarly, the Total Anxiety subscale be excluded from scoring if more than three items are missing.

   c. Reverse scoring: None required

4. *Interpretation*
   a. Clinical thresholds (T-scores):
      i. T-score < 65: Normal
      ii. T-score 65-69.9: Borderline clinical threshold
      iii. T-score ≥ 70: Clinical threshold
   b. Psychometric properties [38]:
      i. Internal consistency: Cronbach's α is generally good to excellent, typically ranging from 0.80 to 0.96 across total and subscale scores.
      ii. Test-retest reliability: Exceeds benchmarks for good reliability (ICC = 0.73 for anxiety, 0.70 for depression)
      iii. Criterion validity:
         (a) AUC = 0.79 for anxiety
         (b) A cutoff score of ≥ 12 corresponded to a sensitivity of 0.92 and a specificity of 0.65
         (c) A cutoff score of ≥ 14 corresponded to a sensitivity of 0.75 and a specificity of 0.76
   c. Direction of scoring: Higher T-scores indicate greater symptom severity
5. *Licensing/permissions*: The RCADS-25 is free to use under a limited license provided by developers Bruce F. Chorpita and Susan H. Spence, available directly from the official RCADS UCLA Website.

C. Perceived Stress Scale for Children (PSS-C)

1. *General Description*
   a. Full name: Perceived Stress Scale for Children (PSS-C)
   b. Original author(s): White B (2014) [39], adapted from Cohen et al. (1983) [42]
   c. Construct measured: Children's subjective perceived stress (global appraisal of stress rather than specific stressors)
   d. Target population: Children and adolescents aged 5-18 years
   e. Respondent: Child/adolescent self-report
   f. Number of items: 13
   g. Recall period: Past week ("last week")
2. *Administration*
   a. Format: Paper-and-pencil or electronic self-report; uses a visual Likert scale appropriate for younger children
   b. Estimated completion time: ~5 minutes

c. Response options: 4-point Likert scale: 0 = "Never," 1 = "A little," 2 = "Sometimes," 3 = "Often"
d. Special instructions: Questionnaire is answered independently by the participant with research staff available for questions.
e. Training requirements: Minimal; may require adult guidance for younger children (ages 5-7)

3. *Data Processing / Scoring*
   a. Scoring algorithm:
      i. Sum raw scores for all 13 items.
         (a) Standard Score for Negative Items: 1, 3, 4, 7, 8, 11
         (b) Reverse Score for Positive Items: 2, 5, 6, 9, 10, 12, 13
      ii. Total score range: 0-39
   a. Handling of missing data: The PSS-C [39] does not provide explicit published guidance on handling missing items. Based on best practices, ≤ 20% missing items is allowable.
2. *Interpretation*
   a. Clinical cutoffs: PSS-C is used as a continuous measure of perceived stress.
   b. Psychometric properties [43]:
      i. Internal consistency: Cronbach's α ranged from 0.52 to 0.84 with a mean of 0.73
   c. Direction of scoring: Higher scores indicate greater perceived stress
3. *Licensing/permissions*: The standard Perceived Stress Scale (PSS) and its variations like the PSS-Child (PSS-C) are generally free for non-profit academic research and educational use without formal written permission. For commercial, corporate, or funded clinical trials, you must secure a license and pay user fees through authorized distributors like MAPI Research Trust ePROVIDE (Lyon, France).

D. Children's Report of Sleep Patterns - Sleepiness Scale (CRSP-S)

1. *General Description*
   a. Full name: Children's Report of Sleep Patterns - Sleepiness Scale (CRSP-S)
   b. Original author(s): Meltzer L.J. et al. (2012) [40]
   c. Construct measured: Self-reported daytime sleepiness in school-aged children
   d. Target population: Children aged 8-12 years (validated also for adolescents 13-18 years via the broader CRSP)
   e. Respondent: Child/adolescent self-report
   f. Number of items: 5
   g. Subscales: Unidimensional (single sleepiness factor)
   h. Recall period: No fixed timeframe specified

2. *Administration*
    a. Format: Paper-and-pencil or electronic self-report
    b. Estimated completion time: ~5 minutes
    c. Response options: 5-point Likert scale: 1 = “Never”, 2 = “Not very often”, 3 = “Sometimes”, 4 = “Usually”, 5 = “Always”
    d. Special instructions: Questionnaire is answered independently by the participant with research staff available for questions.
    e. Training requirements: Minimal
3. *Data Processing / Scoring*
    a. Scoring algorithm: Sum of all completed items.
    b. Handling of missing data: The CRSP-S [40] does not provide explicit published guidance on handling missing items. Based on best practices and due to the small number of items, partial completion would not be included in a final analysis.
    c. Reverse scoring: None required
4. *Interpretation*
    a. The scale is used as a continuous measure. Scores range from 5 to 25.
5. *Psychometric properties:*
    a. Internal consistency: Cronbach's α = 0.77
    b. Test-retest reliability: r = 0.82
    c. Validity: Construct and convergent validity demonstrated with actigraphy, parental reports, and clinical group comparisons
    d. Direction of scoring: Higher scores indicate greater daytime sleepiness
6. *Licensing/permissions*: Freely accessible for non-commercial educational, clinical, and research screening purposes when properly referenced.
